# Trauma- and stressor-related disorders among hematological cancer patients with and without stem cell transplantation: protocol of an interview-based study according to updated diagnostic criteria

**DOI:** 10.1186/s12885-019-6047-9

**Published:** 2019-09-02

**Authors:** Peter Esser, Katharina Kuba, Jochen Ernst, Anja Mehnert-Theuerkauf

**Affiliations:** Department of Medical Psychology and Medical Sociology, University of Leipzig, Philipp-Rosenthal-Str. 55, 04103 Leipzig, Germany

**Keywords:** Trauma- and stressor-related disorders, Hematological malignancies, Survivorship, Stem cell transplantation, Patient-reported outcomes, Quality of life

## Abstract

**Background:**

Trauma- and stressor-related disorders pose an important threat for patients with medical conditions by negatively affecting the outcomes of the underlying somatic disease. Nevertheless, research on distress in the course of hematological cancer is sparse to date. For this patient group, however, treatment is often more toxic and invasive than for other cancer populations. A subgroup of these patients is treated with stem cell transplantation (SCT) which is associated with many stressors including spatial isolation or fear of life-threatening complications. Existing results are inconsistent and primarily based on self-report questionnaires and small samples. Moreover, diagnostic criteria of trauma- and stressor-related disorders have recently been updated.

**Methods:**

This German cross-sectional study will recruit at total of 600 hematological cancer patients, of which 300 will have undergone either autologous or allogeneic SCT. Participants will be assessed for trauma- and stressor-related disorders (adjustment disorder and posttraumatic stress disorder) using a structured clinical interview (SCID-5) based on updated diagnostic criteria. Qualitative investigation of the reported stressors will be used for differential diagnostic investigations and to examine which stressors are experienced as most distressing. Additionally, severity of distress (i.e., general distress as well as anxious, depressive and stressor-related symptomatology) will be assessed by validated questionnaires. We will (i) provide the prevalence of trauma- and stressor-related disorders, (ii) investigate medical and sociodemographic risk factors and (iii) compare the levels of distress within the patient group (SCT vs. non-SCT) and between patients and age- and gender-matched reference groups from the German general population.

**Discussion:**

This study will assess the prevalence of stressor-related disorders and the level of distress among hematological cancer patients across different treatment settings. Identification of medical and sociodemographic risk factors will help to closely monitor patients with a high risk of distress and to deliver psycho-oncological treatment as soon as possible. Comparisons between patients and norm values will be used to identify the need for psycho-oncological treatment in subgroups of hematological patients and thus help to further develop and implement tailored psycho-oncological interventions.

**Electronic supplementary material:**

The online version of this article (10.1186/s12885-019-6047-9) contains supplementary material, which is available to authorized users.

## Background

Disorders which are supposed to develop as a direct consequence of a certain stressor including posttraumatic stress disorder (PTSD) and adjustment disorder (AD) have been recently summarized in a category named *trauma- and stressor-related disorders* [1, 2]. Empirical findings demonstrate the detrimental impact of trauma- and stressor-related disorders among patients with physical conditions such as decreased use of medical health services, worse compliance or poorer treatment outcomes [3–5]. In the long-term, an untreated PTSD in the course of a somatic disease may be even more stressful than the underlying disease itself [6].

To date, a relatively large body of research has focused on traumatic or severely distressing events in the course of a cancer disease [5]. Nevertheless, few studies have investigated this issue among hematological cancer patients, a heterogeneous group for which treatment is often more invasive and toxic than for other oncological populations [7]. A subgroup of hematological cancer patients even has to undergo stem cell transplantation (SCT) receiving stem cells from either the own body (autologous) or a donor (allogeneic) [8]. Even though SCT is considered the only potential cure in many cases [9], patients are at a high risk of life-threatening infections [8] and thus need to stay in spatial isolation [10]. In the case of *allogeneic* SCT, life-threatening rejection reactions against the donor cells (GvHD) may occur affecting all parts of the body including skin, liver or the gastrointestinal tract [11]. Despite considerable improvements in therapy, the mortality rate caused by such complications is still high [8]. Facing all these threats for health and life may lead to high levels of anxiety and uncertainty in this patients group, which in turn is shown to significantly contribute to post-traumatic stress symptomatology [12]. Taken together, undergoing SCT may be considered a severely distressing, potentially traumatic experience in a patient’s life [13].

The current state of research on stressor-related symptomatology among hematological cancer patients is limited: Prevalence rates of PTSD across the few relevant studies range between 8 and 14 % [14–16]. Prevalence rates of PTSD in SCT patients are ranging from 5 to 19% [17]. Except for one study [18], however, these rates are based on self-report measurements and most studies investigated relatively small samples (*N* < 100). Furthermore, the diagnostic criteria for stressor-related disorders have recently been updated (DSM-5). As a result, adjustment disorder (AD) may gain importance as an adequate diagnosis to describe severe stressor-related symptomatology in cancer patients [19, 20]. To date, it remains unclear whether PTSD or AD may be more frequent in cancer populations [19]: Even though a large study (*n* = 2141) showed PTSD to be less frequent than AD (2.0% [21] vs. 12.4% [22]), these results were based on different cancer entities and old dagnostic criteria. For the specific population of patients with SCT, we could identify only one study assessing AD [23]; to our knowledge, studies that assess both AD and PTSD in this patient group do not exist yet. To date, it also remains unclear whether stressor-related symptoms in cancer patients are indicators of a trauma- and stressor-related disorder, or another mental disorder that symptomatically overlaps with PTSD or AD [19]. Such differential diagnostic issues are important for an accurate diagnosis of emotional distress in cancer patients in order to provide adequate psycho-oncological care [19].

Two previous studies (*n* = 886 and *n* = 107) identified sociodemographic and medical risk factors for stressor-related symptomatology in hematological cancer patients and found associations with a variety of sociodemographic (e.g., younger age and employment status) and medical variables (e.g., disease status) [15, 16]. For the specific subgroup of patients with SCT, interpretation of previous findings is limited: Whereas some identified risk factors (e.g., history of a mental disorder) were similar to those found in other (non-cancer) populations [17], gender does not seem to affect stressor-related symptomatology in this population [17]. Results on other risk factors such as level of education, disease stage and hospital stay are still inconsistent or need to be replicated [17]. To date, the impact of SCT in general and the type of SCT (allogeneic vs. autologous) on stressor-related symptomatology remains unclear given that most previous studies did not control for important confounders such as remission status or comorbidity [24]. Such information, however, would be highly needed to tailor psycho-oncological interventions for subgroups in the hematological setting [25].

Research comparing levels of distress (i.e., general distress as well as depressive, anxious or stressor-related symptomatology) among hematological cancer patients with respective norm values is sparse to date. Most of these studies focus on specific subtypes (e.g., [26, 27]), which hampers a comparison across different diagnostic subgroups. One study compared different subsamples of hematological cancer patients (*n* = 922) with norm values and found that all subsamples were meaningfully impaired [28]. Even though patients with SCT did not statistically differ from those without SCT, SCT patients scored worse on all outcomes [28]. Nevertheless, this study investigated quality of life, which may not be generalized to stressor-related symptomatology [28].

### Research objectives

In order to fill existing research gaps and to overcome some of the methodological limitations in previous studies, we will assess 600 hematological cancer patients (300 *with* SCT and 300 *without* SCT) by diagnostic interviews and a battery of validated questionnaires. Our study aims to answer the following research questions:

1. Prevalence: What is the prevalence of trauma- and stressor-related disorders (PTSD and AD) according to updated diagnostic criteria?
2. Risk factors: What are medical and sociodemographic risk factors for trauma- and stressor-related symptomatology?
3. Impairments: Does the level of distress (i.e., general distress as well as anxious, depressive and stressor-related symptomatology) in patients differ from age- and gender-matched reference groups? Does the level of distress in patients with SCT differ from patients without SCT?

Given the inconsistencies in previous research, detailed hypotheses do not seem feasible. Based on the few findings, however, we hypothesize that the prevalence of PTSD will be less frequent than the prevalence of AD (research question 1). We further hypothesize that stressor-related symptomatology will not be associated with gender, but with a history of a previous trauma or mental disorder (research question 2). We finally hypothesize that patients will report higher levels of distress than the general population, and that patients with SCT report higher levels of distress when compared to patients without SCT (research question 3).

Our findings will provide prevalence and severity of stressor-related symptomatology among hematological patients across different treatment settings. Differential diagnostic analyses, e.g., by investigating whether reported intrusions are in fact related to events in the past or represent rather future-oriented ruminations, will allow appropriate diagnosis and thus provide the basis for effective treatment of highly distressed cancer patients. Knowledge on risk factors will enable clinicians to closely monitor patients at high risk of severe psychological distress in order to offer adequate psycho-oncological treatment as soon as possible. Comparison between patients and normative values as well as within the patient group (SCT vs. non-SCT) will investigate the significance, size and practical meaning of impairments in different subgroups of hematological patients and thus may serve as important evidence to establish and implement tailored treatment programs for these populations.

## Methods/design

The study protocol complies with the STROBE guidelines. The STROBE checklist can be found as supplementary file (Additional file 1).

### Study design

**Fig. 1:**
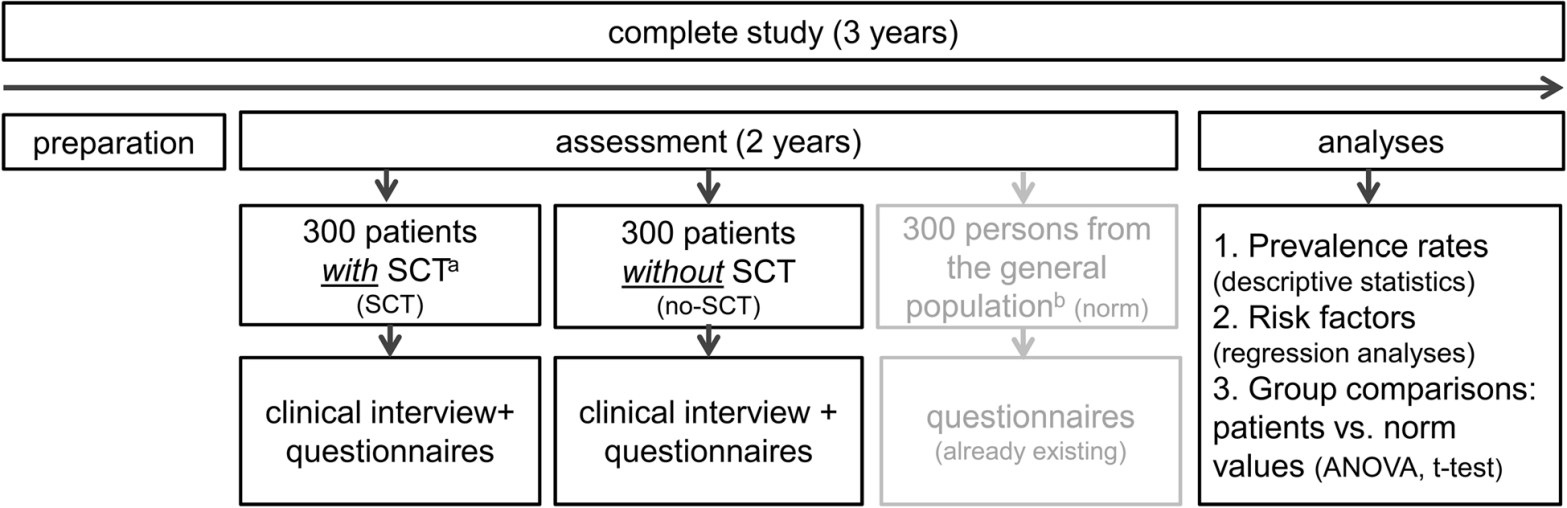
Study Design. ANOVA, analyses of variance; allo-SCT, allogeneic SCT; auto-SCT, autologous. SCT. ^a^ we aim to achieve an even proportion between autologous and allogeneic SCT. ^b^ gender- and age-matched comparison groups will be drawn from large original data sets

We will conduct a cross-sectional study assessing stressor-related disorders/symptomatology in 600 hematological cancer patients, of which 300 will have undergone SCT (Fig. 1). The study will be carried out by the Department of Medical Psychology and Medical Sociology at the University of Leipzig. Patients will be assessed by a structured clinical interview and validated questionnaires 6–8 weeks after the end of treatment. This time point was considered to be (i) as close as possible to the potentially traumatic events and (ii) far enough from treatment to ensure that the (majority of) potential stressors in the course of the therapy has already ended. In order to compare the levels of distress of patients with norm values, gender- and age-matched reference groups from the general population will be generated.

### Study participants

This study will assess patients with (i) malignant neoplasms of lymphoid, hematopoietic and related tissue (ICD-10: C81-C96), (ii) a minimum age of 18 years, (iii) maximum age of 70 years, (iv) cognitive ability to be able to give informed consent for study participation, (v) fluency in German to complete the interview and the questionnaires and (vi) no plans for re-admission at the time of study assessment. Of the 600 participants, 300 will have undergone SCT (allogeneic or autologous). Among patients with SCT, we try to achieve an even proportion between autologous and allogeneic SCT patients.

### Recruitment

Patients in both groups (SCT and non-SCT) will be consecutively recruited over 2 years in the collaborating Department of Hematology of the University Medical Center of the city of Leipzig. Patients are asked by their treating physicians for their consent to be approached by the study team. In case they refuse to be contacted, patients are asked to provide basic data for responder analyses (reason for denial, age, gender, diagnosis and treatment). Patients who agree to be contacted will be approached by a study member during his/her hospital stay (inpatients)/ambulatory visits (outpatients) to provide detailed information of the study. Patients who finally agree to participate in the study will sign the declaration of consent prior to assessment.

### Minimum sample size

One of the major objectives of this study is to provide prevalence rates for post-traumatic stress disorder (PTSD) and adjustment disorder (AD) among hematological cancer patients with and without SCT. For determining the minimum sample size that is needed to adequately answer this research question, we used an algorithm of the World Health Organization that includes (i) the anticipated prevalence in the target population and (ii) the intended precision of the empiric prevalence rate [29]. With respect to anticipated prevalence rates, previous studies among cancer populations point to rates up to 5% (PTSD) [18] and 23% (AD) [23]. Regarding precision, we determined the prevalence estimation to be within a 95%-confidence interval of no more than 5% points. Combining these two parameters, we have to recruit at least 73 patients for prevalence estimates for PTSD, and at least 288 patients to estimate rates for AD. Therefore, a sample size of 300 patients for each group (SCT and non-SCT) seems reasonable, resulting in a total sample of ***N*** **= 600 patients**.

### Feasbility

A member of our working group (AMT) has succesfully conducted a study with similar assessment and recruitment procedure [30], in which the response rate was almost 70%. Based on this experience, we will need to approach about 429 patients for each group (SCT and non-SCT) to achieve the minimum number of 300 participants. Based on patients statistics in our colloborating Department of Hematology, there will be enough eligible patients who can be approached within the recruitment phase. Furthermore, we have planned an additional recruitment buffer of six months in case the response rate will be too low.

### Comparison groups

Thanks to surveys undertaken by our department together with a demographic consulting company (USUMA, Berlin, Germany), the study group has access to large data sets containing nationwide and randomly selected samples among the general adult German population. In detail, we have original data for quality of life measured with the EORTC QLQ-C30 (*N* = 2448) [31], general psychological distress measured with the NCCN Distress Thermometer (*N* = 2437; not published yet), generalized anxiety symptomatology measured with the GAD-7 (*N* = 5030) [32], depressive symptomatology measured with the PHQ-9 (*N* = 5018) [33] and posttraumatic stress disorder symptomatology according to the updated criteria measured with the PCL-5 (*N* = 2500; not published yet).

### Assessment

Patients will be assessed by a clinical interview (SCID-5) and a set of validated self-report questionnaires. About 6–8 weeks after end of treatment, the patients will be mailed the questionnaires in one single paper-pencil document (labeled as “study questionnaire”) to be filled out at home. Having completed the questionnaire, it can be sent back via a pre-stamped envelope. In addition to the questionnaire, patients will be contacted by phone to arrange a meeting for the clinical interview which will be conducted by a trained study member. This interview can be conducted either in person in the study institution or by phone. There will be a maximum time of 14 days between the mailing of the questionnaire and the assessment via the clinical interview. Each assessment (questionnaire and interview) will take between 30 and 60 min. Data for the comparison groups from the general population already exists.

The primary outcomes of this study are trauma- and stressor-related disorders (PTSD, AD), which will be assessed both by interviews and via self-report questionnaires. To comprehensively assess the distress in this patient group, we will also collect mental disorders and types of psychological distress which symptomatically overlap with trauma- and stressor-related symptomatology, such as depressive and anxious symptomatology or fear of recurrence. With respect to questionnaires, we chose instruments which were short, validated in German and used worldwide to ensure comparability with other studies. The assessments are summarized in Table 1, a detailed description of each instrument can be found below. Apart from one exception, all questionnaires used in this study have already been published elsewhere (for sources see Table 1 or the respective paragraphs in the manuscript). The only assessment which was developed in our department (to collect sociodemographic data) has been translated into English and uploaded as a supplementary file (Additional file 2).

**Table 1:** Outcomes and respective assessment instruments

Outcomes	Instruments	SCT^a^	no-SCT^b^	norm^c^
General information				
Sociodemographic	Standard inventory	X	X	X
Medical	Medical chart	X	X	
Self-report questionnaires				
Depressive symptomatology	PHQ-9 [34]	X	X	X
Anxious symptomatology	GAD-7 [34]	X	X	X
General psychological distress	Distress Thermometer [35]	X	X	X
AD symptomatology	ADNM-20 [36]	X	X	
PTSD symptomatology	PCL-5 [37]	X	X	X
Posttraumatic growth	PTGI [38]	X	X	
Experiential Avoidance	BEAQ [39]	X	X	
Fear of progression	PA-F-SF [40]	X	X	
Quality of life	EORTC-QLQ-C30 [41]	X	X	X
Comorbidity	CI [42]	X	X	
Clinical interview	SCID-5 [43]			
Pre-morbid mental disorders		X	X	
Previous traumata		X	X	
Detailed trauma-list		X	X	
Posttraumatic stress disorder		X	X	
Adjustment disorder		X	X	
Panic disorder		X	X	
Generalized anxiety disorder		X	X	
Major depression		X	X	
Dysthymia		X	X	

*AD* adjustment disorder; *PTSD* posttraumatic stress disorder

^a^ hematological cancer patients *with* stem cell transplantation

^b^ hematological cancer patients *without* stem cell transplantation

^c^ general population (data already exists)

### Description of assessment instruments

#### Clinical interview to assess mental disorders (SCID-5)

The structured clinical interview for DSM-5 (SCID-5) [43] is an internationally used and well-established diagnostic tool to assess the whole spectrum of mental disorders based on the updated criteria of the Diagnostic and Statistical Manual of Mental Disorders published by the American Psychiatric Association (DSM-5). The interview can be conducted after being trained by an expert. In our study, we will assess a large spectrum of mental disorders including trauma- and stressor-related disorders (see Table 1).

#### Sociodemographic and medical information

Sociodemographic variables such as gender, age, family status or education are assessed via a standard module developed in the study center. An English translation of this assessment has been uploaded as a supplementary file (Additional file 2).

Medical data including diagnosis according to ICD-10, information about current treatment (type, frequency and last date of therapy), previous treatments (including previous SCT) and respective dates, disease status (remitted vs. non-remitted), total time of hospital stay, total time in isolation in the course of SCT, history of relapse, any previous malignancy, occurrence of acute GvHD and level of physical functioning (Karnofsky-Index) are obtained from the medical chart.

#### Quality of life (EORTC QLQ-C30)

We will use the European Organization for Research and Treatment of Cancer Quality of Life Questionnaire (EORTC QLQ-C30) [41, 44]. The questionnaire contains 30 items which form five functioning scales (physical, role, cognitive, emotional and social), three symptom scales (fatigue, nausea, pain), six one-item scales (dyspnea, sleeping problems, loss of appetite, constipation, diarrhea, financial problems) and one global scale (including general health and global quality of life).

#### Depressive and anxious symptomatology (PHQ-9 and GAD-7)

The Patient Health Questionnaire (PHQ) [34, 45] assesses mental disorders according to DSM-IV criteria by measuring the frequency of respective symptoms within the last two weeks. For our study, we will assess depressive (9 items, PHQ-9) and anxious (7 items, GAD-7) symptomatology.

#### General distress (distress thermometer)

The distress thermometer of the *National Comprehensive Cancer Network* (NCCN) measures general distress in cancer patients [35, 46]. The instrument consists of a single-item visual analogue scale ranging from 0 (no distress) to 10 (extremely distressed).

#### Fear of progression (FoP-Q-SF)

The Short Form of the German Fear of Progression Questionnaire (FoP-Q-SF) has been developed for chronically ill patients [40]. This 12-item questionnaire assesses fear of progression on 4 dimensions (affective reactions, partnership/family, work, loss of autonomy).

#### Comorbidity (CI)

We will use a validated comorbidity assessment instrument [42]. This questionnaire assesses a variety of conditions and the degree to which they interfere with daily activities. We translated this instrument into German and adapted it to hematological cancer patients, i.e., we merged some items (e.g., ‘coronary heart disease’ and ‘congestive heart failure’ to the variable ‘heart diseases’) and added comorbidities which are frequent among hematological cancer patients such as skin problems, mucosal issues, anemia and liver disease. The adapted version assesses 24 comorbidities and has been applied in a previous study [28].

#### Adjustment disorder symptomatology (ADNM-20)

The Adjustment Disorder – New Module 20 (ADNM-20) is a 20-item questionnaire that assesses symptoms of adjustment disorder according to updated diagnostic criteria [36]. Originally, the questionnaire consists of two parts, a stressor list assessing distressing events in the past two years and an item list assessing respective symptomatology in response to each of the experienced stressors. For our purpose, we will omit the stressor list and explicitly ask the patient to report on symptomatology related to cancer- and treatment-related events.

#### Posttraumatic stress disorder (PTSD) symptomatology (PCL-5)

The Posttraumatic stress disorder checklist – fifth edition (PCL-5) is a 20-item questionnaire assessing symptoms of PTSD on four dimensions (intrusion; avoidance; hyperarousal; alterations in cognitions and mood) according to updated diagnostic criteria [37, 47]. For our purpose, we will explicitly ask the patient to report on symptomatology related to cancer- and treatment-related events.

#### Posttraumatic growth inventory (PTGI)

The Posttraumatic Growth Inventory (PTGI) [38, 48] assesses positive outcomes which may occur in persons after the experience of traumatic events. On 21 items, the questionnaire assesses different aspects (new possibilities, relating to others, personal strength, spiritual change, and appreciation of life). For our purpose, we will ask the patient to report changes associated with cancer- and treatment-related events.

#### Experiential avoidance (BEAQ)

We will assess experiential avoidance using the one-dimensional Brief Experiential Avoidance Questionnaire (BEAQ) [39]. On 15 items, participants rate their tendency to avoid unwanted internal experiences such as negative feelings or cognitions on a 6-point Likert scale. The BEAQ has been applied in different study populations [39].

### Statistical analyses

We will investigate the prevalence of trauma- and stressor-related disorders via absolute percentages (based on the data of the clinical interviews).

Given that the case numbers of patients with AD or PTSD may be very low, analyses on risk factors will be conducted using the dichotomous results of the clinical interviews (multiple logistic regressions) and the continous results of the questionnaires (multiple linear regressiosn). All regression analyses which will be controlled for important confounders such as age, gender, time since SCT, comorbidity and disease status.

Group comparisons between patients and norm values will be conducted via t-tests/Mann Whitney U-tests. Comparison groups from the general population will be matched to the patient groups with respect to age and gender. Comparisons between non-SCT and SCT patients will be conducted via analyses of covariance (ANCOVA) in order to control for age, gender and important medical variables such as time since diagnosis and number of previous oncological treatments. In addition to test for significance, we will calculate effect sizes (t-tests: Cohen’s d; ANCOVA: Eta-square) in order to test for the size and thus clinical relevance of group differences. Group effects have to reach at least medium size to be defined as clinically relevant (Cohen’s d ≥ .5; Eta-square ≥ .06).

Depending on the frequency and type of missing data (missing completely at random vs. missing at random vs. missing not at random), listwise deletion of patients with missing data or appropriate imputation techniques will be applied. In case of multiple comparisons among the same sample, signifcance levels will be Bonferroni-adjusted. Analyses will be conducted with SPSS 24 (2011, IBM Corporation, Armonk, USA) and R (The R Foundation of Statistical Computing, 2010).

### Differential-diagnostic analyses

In the course of the assessment of PTSD and AD, we will ask the patient for all kinds of potentially traumatic events as listed in the clinical interview (e.g., severe accident, rape). In addition to this general trauma list, we will ask for the occurrence of specific cancer- and treatment-related stressors. If the patient confirms the occurrence of cancer- and treatment-related stressors, he or she is asked to report on each of these stressors in more detail. Such qualitative information will later be used for differential diagnostic information (e.g., for differentiating PTSD from AD and vice versa): For example, it may be examined whether the necessary trauma criteria are met or whether reported intrusions are related to events in the past or rather represent future-oriented ruminations. This approach may also enable to examine whether the stressor-related symptomatology in patients with SCT is directly related to the SCT or to other cancer-related events. Finally, some side effects of the therapy may mimic stressor-related symptomatology, particularly cognitive symptoms (memory and concentration) and sleep problems. Therefore, we will conduct sensitivity analyses by providing prevalence rates of PTSD without using these potentially confounding symptoms.

### Bias control

Responders and non-responders will be compared in central sociodemographic (age and gender) and medical (type of diagnosis and treatment) characteristics. In case of significant group differences in certain variables, these will be considered as confounders and thus taken into account in statistical analyses and the interpretation of results. We will assess reasons for non-participation to identify additional sample biases (e.g., physical or psychological burden). All comparison samples from the general population will be matched to the patient groups with respect to age and gender. Comparison within the group of patients (SCT vs. non-SCT) will be controlled for differences in sociodemographic and medical characteristics. Additionally, we will conduct sensitivity analyses for subgroups provided that the sample size for these subsets will be large enough.

## Discussion

Research on trauma- and stressor-related symptomatology in hematological cancer patients is sparse and has several methodological limitations which limits the validity of previous findings. We aim to obtain a large sample of hematological cancer patients, of which one half will have undergone autologous or allogeneic SCT. Each patient will be assessed by a structured clinical interview according to updated diagnostic criteria and a set of validated questionnaires. Access to large data sets of the German adult population will help us to generate comparison groups perfectly matched by important sociodemographic variables. Patient recruitment from the clinic ensures us to obtain valid medical data. A limitation of the study will be the cross-sectional design, which does not allow for interpreting results as cause-and-effect relationships. Nevertheless, the information of this study (e.g., with respect to response rates, prevalence rates etc.) is intended to be used to build up a cohort of survivors which are followed for several years.

Our study will help to inform the health care system and health care providers about the specific burden associated with hematological malignancies in general and patients with SCT in particular. Findings will also help to identify highly distressed patients as soon as possible and to provide clinical data to develop and implement tailored supportive care programs for specific subgroups of hematological cancer patients.

## Additional files

Additional file 1: STROBE checklist. (DOC 87 kb)

Additional file 2: Standard Inventory to assess sociodemographic data. (DOCX 21 kb)

## Data Availability

The datasets used and/or analysed during the current study will be made available from the corresponding author on reasonable request.

## Abbreviations

AD: Adjustment disorder
ANCOVA: Analysis of covariance
DSM: Diagnostic and Statistical Manual of Mental Disorders
GvHD: Graft-versus-Host-Disease
ICD: International Classification of Diseases
PTSD: Posttraumatic stress disorder
SCID: Structured Clinical Interview for DSM
SCT: Stem cell transplantation
