# Supplementary material for: Trauma- and stressor-related disorders among hematological cancer patients with and without stem cell transplantation: protocol of an interview-based study according to updated diagnostic criteria
Source: BMC Cancer. 2019 Sep 2;19:870. doi: 10.1186/s12885-019-6047-9 (PMC6720985; doi:10.1186/s12885-019-6047-9)
Supplement: Supplementary file 2 — Standard Inventory to assess sociodemographic data. (DOCX 21 kb) [file 12885_2019_6047_MOESM2_ESM.docx]

| 1. **Gender** | | | | | | | | | | |
| --- | --- | --- | --- | --- | --- | --- | --- | --- | --- | --- |
|  | | 🔿_0_ female | | 🔿_1_ male | | | 🔿_2_ other | | |  |
| 1. **Marital status** | | | | | | | | | | |
|  | | 🔿_0_ single | | 🔿_1_ married | | | 🔿_2_ divorced | | | 🔿_3_ widowed |
| 1. **Are you in a relationship?** | | | | | | | | | | |
|  | | 🔿_0_ No | 🔿_1_ Yes | | | | | | | |
| 1. **Do you have children (incl. step- and foster children)?** | | | | | | | | | | |
|  | 🔿_0_ No, I don’t have children | | | | | | | | | |
|  | 🔿_1_ Yes, I have _______ children **🡪** How many children live in your household: _______ (number) | | | | | | | | | |
| 1. **What is your highest level of education?** | | | | | | | | | | |
|  | 🔿_0_ elementary or secondary school | | | | | 🔿_1_ middle school or POS | | | | |
|  | 🔿_2_ technical college | | | | | 🔿_3_ university/college | | | | |
|  | 🔿_4_ no degree | | | | | 🔿_5_ other: __________________ | | | | |
| 1. **What is your current labor situation/occupation?** | | | | | | | | | | |
|  | 🔿_0_ full time working | | | | 🔿_1_ part time working | | | 🔿_2_ in education/studies | | |
|  | 🔿_3_ housewife/-husband | | | | 🔿_4_ unemployed | | | 🔿_5_ EU-pension | | |
|  | 🔿_6_ early retirement | | | | 🔿_7_ retirement | | | 🔿_8_ other: ______________ | | |
| 1. **What is your monthly net household income?** | | | | | | | | | | |
|  | 🔿_0_ less than 1.000 € | | | | 🔿_1_ 1.000 - under 1.500 € | | | | 🔿_2_ 1.500 - under 2.000 € | |
|  | 🔿_3_ 2.000 - under 2.500 € | | | | 🔿_4_ 2.500 - under 3.000 € | | | | 🔿_5_ 3.000 - under 3.500 € | |
|  | 🔿_6_ more than 3.500 € | | | |  | | | |  | |
